# miR128-1 inhibits the growth of glioblastoma multiforme and glioma stem-like cells via targeting BMI1 and E2F3

**DOI:** 10.18632/oncotarget.12385

**Published:** 2016-10-01

**Authors:** Zheng-nan Shan, Rui Tian, Min Zhang, Zhao-hua Gui, Jing Wu, Min Ding, Xin-Fu Zhou, Jie He

**Affiliations:** ^1^ Department of Pathology, Anhui Provincial Hospital affiliated to Anhui Medical University and Anhui Provincial Cancer Hospital, Hefei 230031, China; ^2^ School of Pharmacy and Medical Sciences, Faculty of Health Sciences, University of South Australia, Adelaide SA 5000, Australia

**Keywords:** miR128-1, BMI1, E2F3, DNA methylation, glioblastoma multiforme

## Abstract

MicroRNA128-1 (miR128-1), as a brain-specific miRNA, is downregulated in glioblastoma multiforme (GBM) and closely associated with the progression of GBM. However, the underlying molecular mechanism of the downregulation and its role in the regulation of tumorigenesis and anticancer drug resistance in GBM remains largely unknown. In the current study,we found that miR128-1 was downregulated in GBM and glioma stem-like cells (GSCs). Intriguingly, treatment with the DNA methylation inhibitors 5-Aza-CdR (Aza) and 4-phenylbutyric acid (PBA) resulted in miR128-1 upregulation in both GBM cells and GSCs. Either forced expression of miR128-1 or Aza/PBA treatment inhibited tumor cell proliferation, migration and invasion *in vitro*. Moreover, overexpression of miR128-1 inhibited the growth of transplant tumor *in vivo*. BMI1 and E2F3 were found to be direct targets of miR128-1 and downregulated by miR128-1 *in vitro* and *in vivo*. Our results revealed a mechanism of methylation that controls miR128-1 expression in GBM cells and GSCs and indicate miR128-1 could function as a tumor suppressor in GBM by negatively regulating tumor cell proliferation, invasion and self-renewal through direct targeting BMI1 and E2F3. Our findings suggest that DNA methylation inhibitors are potential agents for GBM treatment by upregulating miR-128-1.

## INTRODUCTION

Glioblastoma multiforme (GBM) is the most common and aggressive primary brain tumor in adults and approximately 20,000 new cases are diagnosed in the United States every year. In the past decade, significant advances have been made in the treatment of GBM; however, even with aggressive systematic surgery, radiation and chemotherapy, the median survival of GBM patients remains shorter than 15 months [1]. The aggressive and diffuse infiltrative growth of GBM is the current challenge for the management of GBM patients. It is thus urgent to elucidate the molecular mechanisms underlying the initiation, progression, maturation and maintenance of GBM, and identify novel strategies for the treatment of GBM patients.

Mounting evidence has shown that glioblastoma cells retain features of neural progenitor cells, including self-renewal and the ability to grow as neurospheres in culture [2, 3]. This subpopulation of tumor cells is also known as glioma stem-like cells (GSCs) and believed to give rise to the heterogeneity of GBM cancer cells and contribute to the resistance to currently available anti-tumor therapies. It is believed that novel therapeutics targeting GSCs could be more effective than traditional anti-glioblastoma chemotherapy [4]. However, the precise mechanism of the regulation of the proliferation, survival and maintenance of GSCs remains elusive.

microRNAs (miRNAs) are small noncoding RNAs (19-22 nt) that negatively regulate gene expression at the post-transcriptional level. More than 600 miRNAs have been identified in human cells; Increasing data have demonstrated that miRNAs play vital roles in most biological processes, including apoptosis, proliferation, differentiation, development and the metabolism [5, 6]. miRNA deregulation has been observed in most cancer types and plays an important role in the multiple steps of tumorigenesis by controlling the expression of oncogenes and tumor suppressor genes [7]. In glioblastoma, microRNA deregulation is closely associated with the differentiation, proliferation, invasion and self-renewal of tumor cells [8, 9]. Particularly, expression of the “brain-specific” miRNA miR128-1 is associated with normal brain development [10]. Recently, significant downregulation of miR128-1 expression was found in glioblastomas and associated with aggressive glioblastoma cell growth [11], but the underlying molecular mechanism of the deregulation of miR128-1 in glioblastoma remains largely unknown.

In the present study, we have investigated the regulation of miR128-1 gene transcription and the downstream targets of miR128-1 in GBM and cultured glioblastoma stem-like cells.

## RESULTS

### miR128-1 transfection results in higher miR128-1 levels in GSCs than in glioma cells

**Figure 1:**
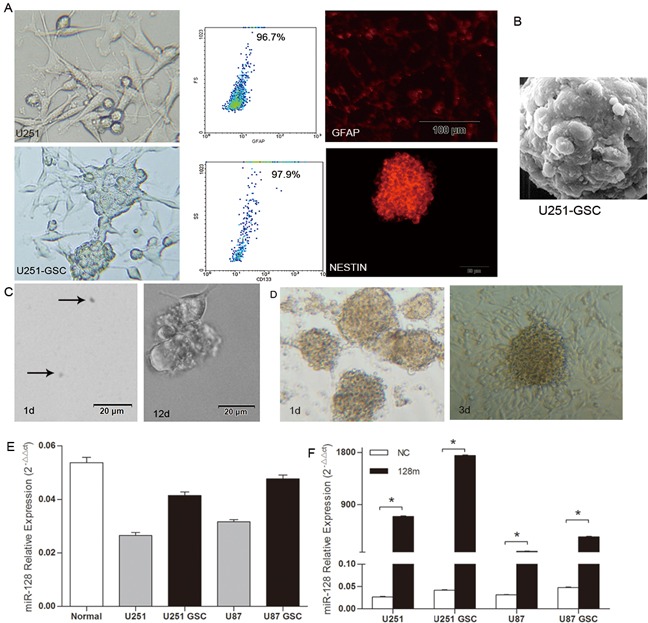
miR128-1 expression was elevated in GSCs when compared to glioma cells **A.** When compared with U251 cells, U251-GSCs showed sphere formation and positive expression of CD133, GFAP and nestin. **B.** Morphological features of GSCs by electron microscopy (magnification, 12000×). **C.** The self-renewal capacity of GSCs as demonstrated by stem cell cloning formation experiments. **D.** The differentiation of GSCs to glioma cells was assessed by differentiation assay. **E.** Low miR128-1 expression in human glioma cell lines U87, U251 and their GSCs when compared with glial cells from the cerebral white matter (normal) as determined by qRT-PCR. Two-tailed unpaired t test: U251 vs normal: t=20.46, *p<0.001; U251-GSCs vs normal: t=9.007, *p<0.001; U87 vs normal: t=17.73, *p<0.001; U87-GSCs vs normal: t=4.295, *p<0.05. **F.** Mature miR128-1 expression was measured by qRT-PCR in U251, U87 and their respective GSCs stably transduced with miR128-1 mimic. Two-tailed unpaired t test: U251 NC vs 128m: t=117.3, *p<0.0001; U251-GSCs NC vs 128m: t=350.9, *p<0.0001; U87 NC vs 128m: t=26.47, *p<0.001; U87-GSCs NC vs 128m: t=68.12, *p<0.0001. Values denote the mean ± SEM of three independent assays.

We established GSCs by culturing U87 and U251 cell lines in DMEM/F12 medium containing B27 and relevant growth factors according to the methods as previously described [12]. Gliospheres were formed within 7-10 days and GSC phenotypes, positive expression of CD133, nestin and GFAP were verified using light microscopy, flow cytometry and immuofluorescence microscopy (Figure 1A). GSCs morphological features were further confirmed using scanning electron microscopy (Figure 1B). Stem cell cloning formation experiments demonstrated the self-renewal capacity of GSCs (Figure 1C). In addition, differentiation assay showed the differentiation of GSCs to glioma cells (Figure 1D). These results demonstrated the successful establishment of GSCs derived from U87 and U251 cell lines.

It has been shown that the expression level of miR128-1 is higher in brain than in glioblastoma [13]. To assess the expression status of miR128-1 in glioblastoma cells derived GSCs, we used microarray analysis to compare miR128-1 expression in GSCs with parental U87 and U251 cell lines and normal human brain tissues. When compared to normal brain tissues, miR128-1 expression was downregulated in GSCs as well as the corresponding U87 and U251 cell lines (Figure 1E). Nevertheless, miR128-1 was increased in both U87-GSCs and U251-GSCs when compared with U87 and U251 cells, respectively, suggesting a role for miR128-1 in the maintenance of the stemness of GSCs. Next, we infected U87 and U251 cells and their respective GSCs derivatives with lentiviral vectors containing miR128-1 primary transcripts or negative control DNA (NC). Cells infected with the lentivirus containing miR128-1 mimic showed much higher miR128-1 levels than cells infected with lentivirus containing miR-NC. Surprisingly, miR128-1 levels were 2-3 fold higher after infection in GSCs than in their original cell lines (Figure 1F). These results suggest that miR128-1 expression is either upregulated or that the miR128-1 molecule is more stable in GSCs.

### Methylation inhibitors Aza and PBA increase miR128-1 expression in glioma cells and GSCs

**Figure 2:**
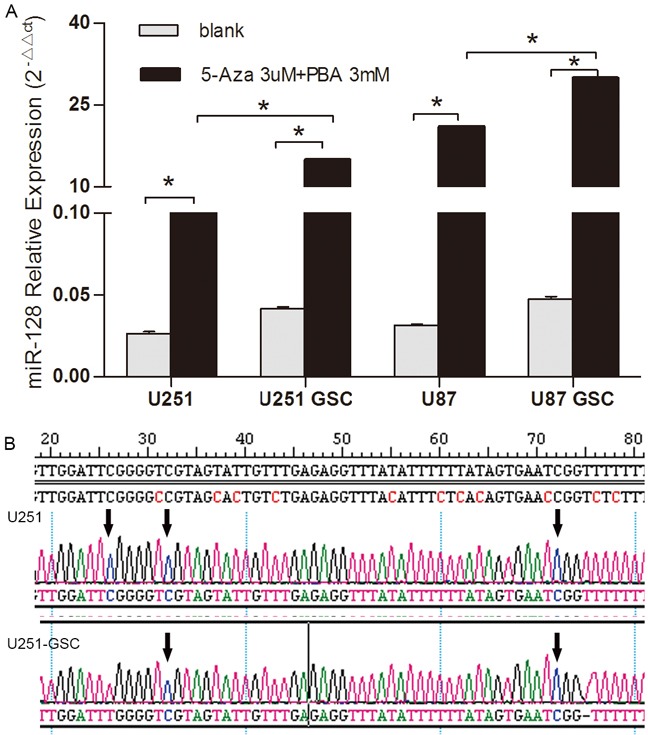
DNA methylation analysis of miR128-1 gene in glioma cells and GSCs **A.** miR128-1 expression was measured by qRT-PCR in U251 and U87 cell lines and their respective GSCs after Aza (3μM) and PBA (3mM) treatment. U251 blank vs 5Aza+PBA: t=126.7, *p<0.0001; U251-GSCs blank vs 5Aza+PBA: t=369.3, *p<0.0001; U87 blank vs 5Aza +PBA: t=335.5, *p<0.0001; U87-GSCs blank vs 5Aza+PBA: t=740.4, *p<0.0001; U251 5Aza+PBA vs U251-GSCs 5Aza+PBA: t=117.4, *p<0.0001; U87 5Aza+PBA vs U87-GSCs 5Aza+PBA: t=119.3, *p<0.0001 (two-tailed unpaired t test). Values denote the mean ± SD of three independent assays. **B.** DNA methylation sites in the miR128-1 gene were identified by bisulfite sequencing PCR (BSP). U251, U87, U251-GSCs and U87-GSCs were tested for three CpG islands whose sequence numbers were 26, 32 and 72. The arrow shows the methylation site which is C site in U251 and U251-GSCs.

Previous studies showed that DNA methylation was correlated with the downregulation of miR128-1 in colorectal cancer [14] and osteoarthritic cartilage [15]. To assess whether DNA methylation plays a role in the downregulation of miR128-1 in glioma cells, we treated glioma U87 and U251 cells and their respective GSCs with the epigenetic methylation inhibitors Aza and PBA. We observed that miR128-1 expression was elevated in all cells following treatment with Aza and PBA (Figure 2A). Furthermore, the increase of miR128-1 expression in GSCs was significantly higher than their respective glioma cell lines. The data suggest that epigenetic methylation might at least in part contribute to miR128-1 downregulation in glioma cells, especially GSCs.

To identify potential methylation sites in the promoter region of the miR128-1 gene, we analyzed the CGIs database to identify CGIs within the miR128-1 gene and found three CGIs at sequence 26, 32 and 72. We next performed bisulfite sequencing PCR (BSP) to assess the methylation status of the miR128-1 gene in U87, U251, U87-GSCs and U251-GSCs. CGIs were found in all cell lines (Figure 2B). Consistent with our observation of higher miR128-1 levels in U87 than U251 cells (Figure 1E), there were one or two methylation sites in U251-GSCs. Impressively, all methylation sites (26, 32 and 72) were present in U87 and U251 cells, consistent with the higher miR128-1 expression in U251-GSCs than in U251 (Figure 1E). Thus, methylation at the 26, 32 and 72 sites may contribute to miR128-1 downregulation in glioma cells.

### miR128-1 directly targets BMI1 and E2F3 in glioblastoma cells

**Figure 3:**
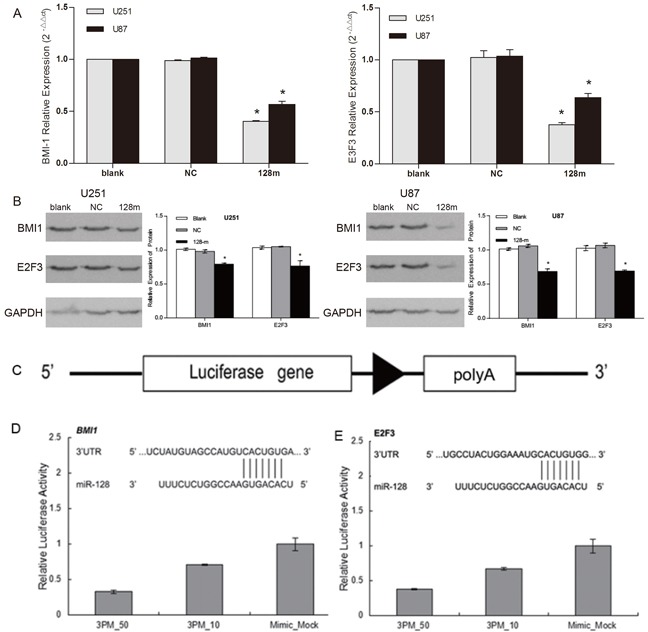
BMI1 and E2F3 were direct targets of miR128-1 **A.** U251 and U87 cells were stably transfected with lentiviral vector expressing miR128-1 or control miRNA (NC). Relative mRNA levels of BMI1 and E2F3 normalized to Actin were measured by qRT-PCR. Each experiment was performed triplicate. One-way analysis of variance: p<0.0001. BMI1 expression in U251: blank vs NC, t=2.492, p>0.05; blank vs 128m, t=143.5, p<0.0001; NC vs 128m, t=141, *p<0.0001. BMI1expression in U87: blank vs NC, t=0.9005, p>0.05; blank vs 128m, t=29.34, *p<0.001; NC vs 128m, t=30.24, *p<0.001. E2F3 expression in U251: blank vs NC, t=0.7823, p>0.05; blank vs 128m, t=19.77, *p<0.001; NC vs 128m, t=20.55, *p<0.001. E2F3 expression in U87: blank vs NC: t=1.085, p>0.05; blank vs 128m, t=10.50, *p<0.01; NC vs 128m, t=11.59, *p<0.001 (Bonferroni's Multiple Comparison Test). **B.** The protein levels of BMI1 and E2F3 in U251 and U87 cells, and those stably transfected with lentiviral vector expressing miR128-1 or NC were assessed by Western blots with GAPDH as loading control. Densitometry analyses showed the relative BMI1 and E2F3 protein levels to GAPDH. miR128-1 mimics significantly reduced both BMI-1 and E2F3 (*P<0.05). **C – E.** Dual luciferase assay in U87 cells. Schematic diagram of the BMI1 and E2F3 3′-UTR reporter construct **(C).** Luciferase activity was measured as relative activity to the corresponding normal control (NC) (mock, assigned as value “1”). Values denote the mean ± SEM of three independent assays.

A number of cell type-dependent miR128-1 potential downstream target genes have been reported, including but not limited to BMI1, CSF1, KLF4, LIN28A, NANOG, SNAIL and E2F3 [16]. BMI1, a component of the PRC2 polycomb repressor complex, has emerged as the most important player for the self-renewal and malignant transformation of glioma [17, 18]. In silico analyses predicted highly conserved binding sites in the 3′-UTR of BMI1 (positions 480 - 488) and 3′-UTR of E2F3 (positions 2039 - 2045) for miR128-1 [16, 19]. To confirm miR128-1 targeting of BMI1 and E2F3 in glioblastoma cells, we measured BMI1 and E2F3 expression in U251 and U87 cells after miR128-1 transfection. Significant decrease in BMI1 and E2F3 mRNAs was observed in both U251 and U87 cells following transfection of miR128-1 mimics (Figure 3A). Consistent with the downregulation of mRNAs, transfection of miR128-1 mimics significantly reduced BMI1 and E2F3 protein levels in both U251 and U87 cells (Figure 3B). To further support the observation that BMI1 and E2F3 are direct targets of miR128-1, dual luciferase assays were performed using glioma cells transfected with BMI1 and E2F3 reporter constructs with or without miR128-1 (Figure 3C). As expected, luciferase activity in glioma cells was reduced by co-transfection of the BMI1 construct and miR128-1, while mutation of BMI1's 3′-UTR miR128-1 binding sites abrogated reduction of luciferase activity by miR128-1 (Figure 3D). Similar results were observed after co-transfection of the E2F3 construct and miR128-1 (Figure 3E). Taken together, these data indicate that miR128-1 directly targets BMI1 and E2F3 in glioblastoma cells.

### miR128-1 overexpression suppresses the proliferation, migration and clonogenicity of glioblastoma cells *in vitro*

**Figure 4:**
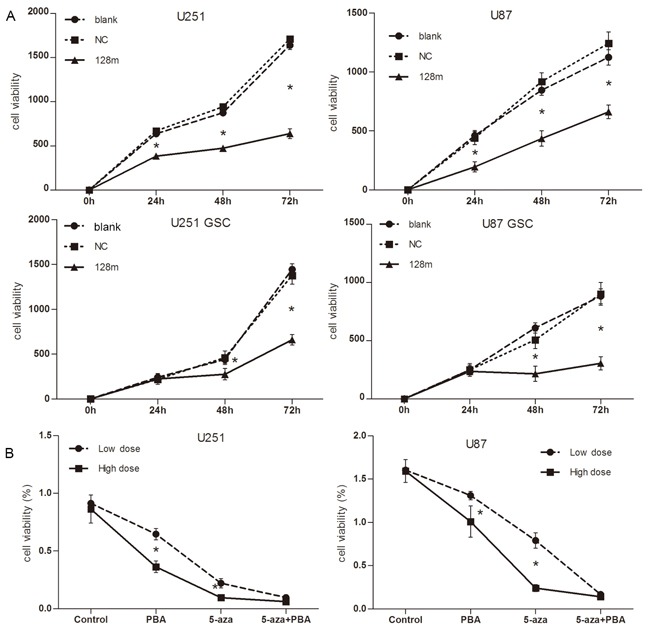
miR128-1 overexpression suppressed cell proliferation *in vitro* **A.** Cell proliferation as detected by CCK-8 assay. The proliferation rate of cells was determined by measurement of absorbance excitation at 450 nm and emission at 600nm using a spectrophotometer. Significant inhibition of cell growth was observed in cells with miR128-1 overexpression when compared to cells transfected with control. Upper panel: U251 and U87; Bottom panel: U251-GSCs and U87-GSCs. Blank: no transfected cells; NC: transfected with control miRNA. Values presented as mean ± SD from triplicate wells. U251, U87, U251-GSCs and U87-GSCs: blank vs NC, p>0.05; blank vs 128m, *p<0.01-0.001; NC vs 128, *p<0.01-0.001 (One-way analysis of variance, *p<0.01-0.001; Bonferroni's Multiple Comparison Test). **B.** Cell proliferation was inhibited by Aza and PBA as measured by CCK-8 assay. U251 and U87 cells were treated with low doses (1 μM, 1 mM) or high doses (3 μM, 3 mM) of Aza and PBA (respectively) for 6 days.

**Figure 5:**
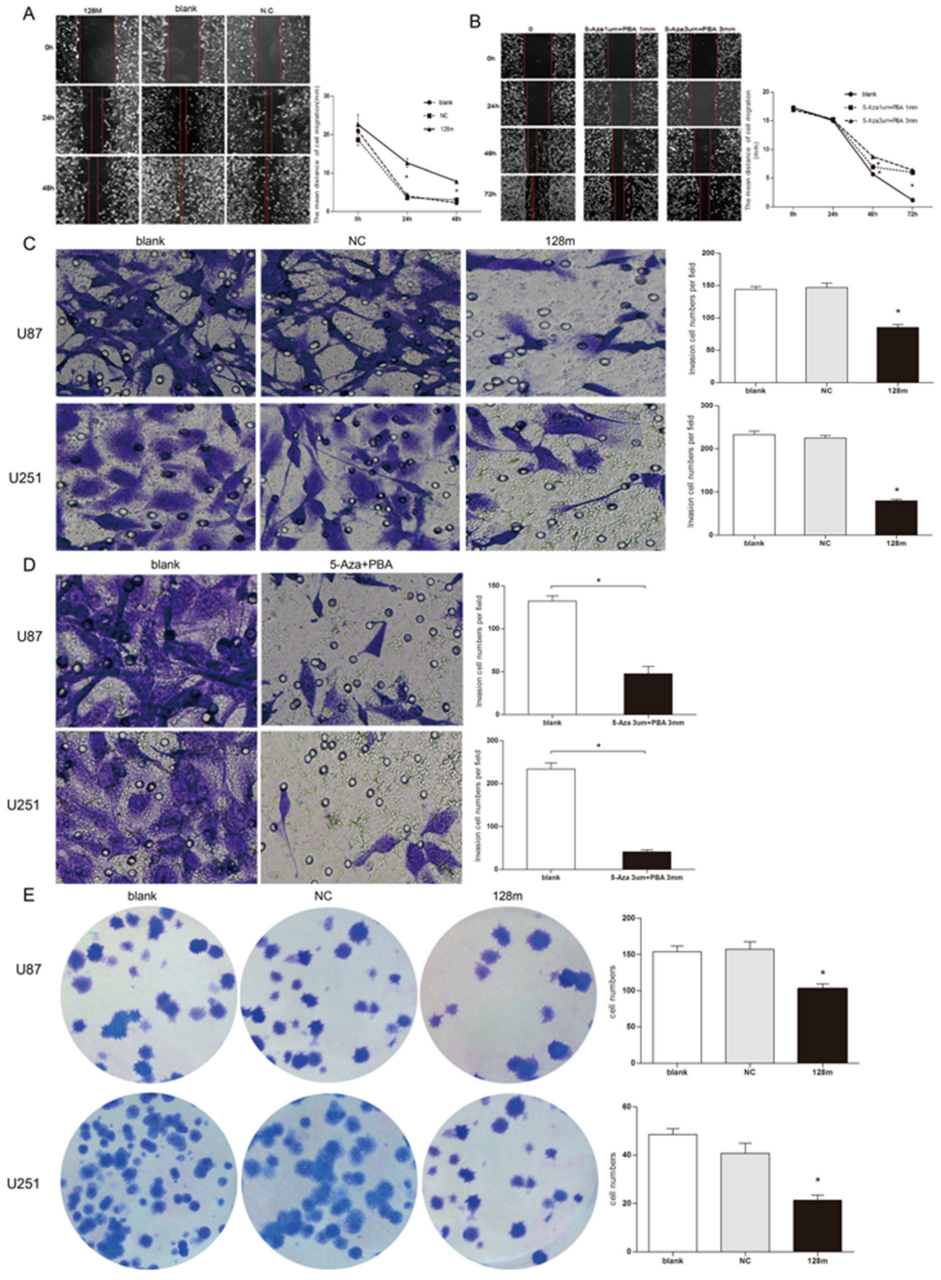
miR128-1 overexpression suppressed migration, invasion and clonogenesis of glioma cells Wound-healing assay of U251 cells transfected with miR128-1 mimic or miR-NC **A.** or after treatment with Aza and PBA **B.** Transwell invasion assay of U251 and U87 cells transfected with miR128-1 or miR-NC **C.** or treated with Aza and PBA **D.**. Soft agar colony formation assay was performed using U251 and U87 cells **E.** transfected with miR128-1 or miR-NC. Representative experiments are shown in triplicate along with the mean ± SEM. *p < 0.01-0.001.

We next assessed whether miR128-1 overexpression would have an effect on the cell proliferation, migration and colony formation of cultured glioma cells and GSCs. Transfection of miR128-1 for 48 hrs apparently decreased the proliferation of glioma U251 and U87 cells when compared with controls (Figure 4A, upper panel). Similarly, the proliferation of U251-GSCs and U87-GSCs was obviously reduced by miR128-1 transfection (Figure 4A, lower panel). Consistent with miR128-1 suppression by methylation, U251 and U87 cells treated with combination of Aza and PBA displayed reduced cell growth (Figure 4B). Similarly, miR128-1 overexpression (Figure 5A) or treatment with 3 μM of Aza plus 3 mM of PBA (Figure 5B) inhibited wound healing in U251and U87 cells. In addition, miR128-1 overexpression (Figure 5C) or treatment with 3 μM of Aza plus 3 mM of PBA (Figure 5D) significantly reduced the cell migration ability of U251 and U87 cells. Furthermore, colony formation assays demonstrated that transfection of miR128-1 significantly decreased the colony formation in both U251 and U87 cells when compared with that of NC and blank control (Figure 5E).

### Overexpression of miR128-1 inhibits the growth of mouse glioma xenografts

**Figure 6:**
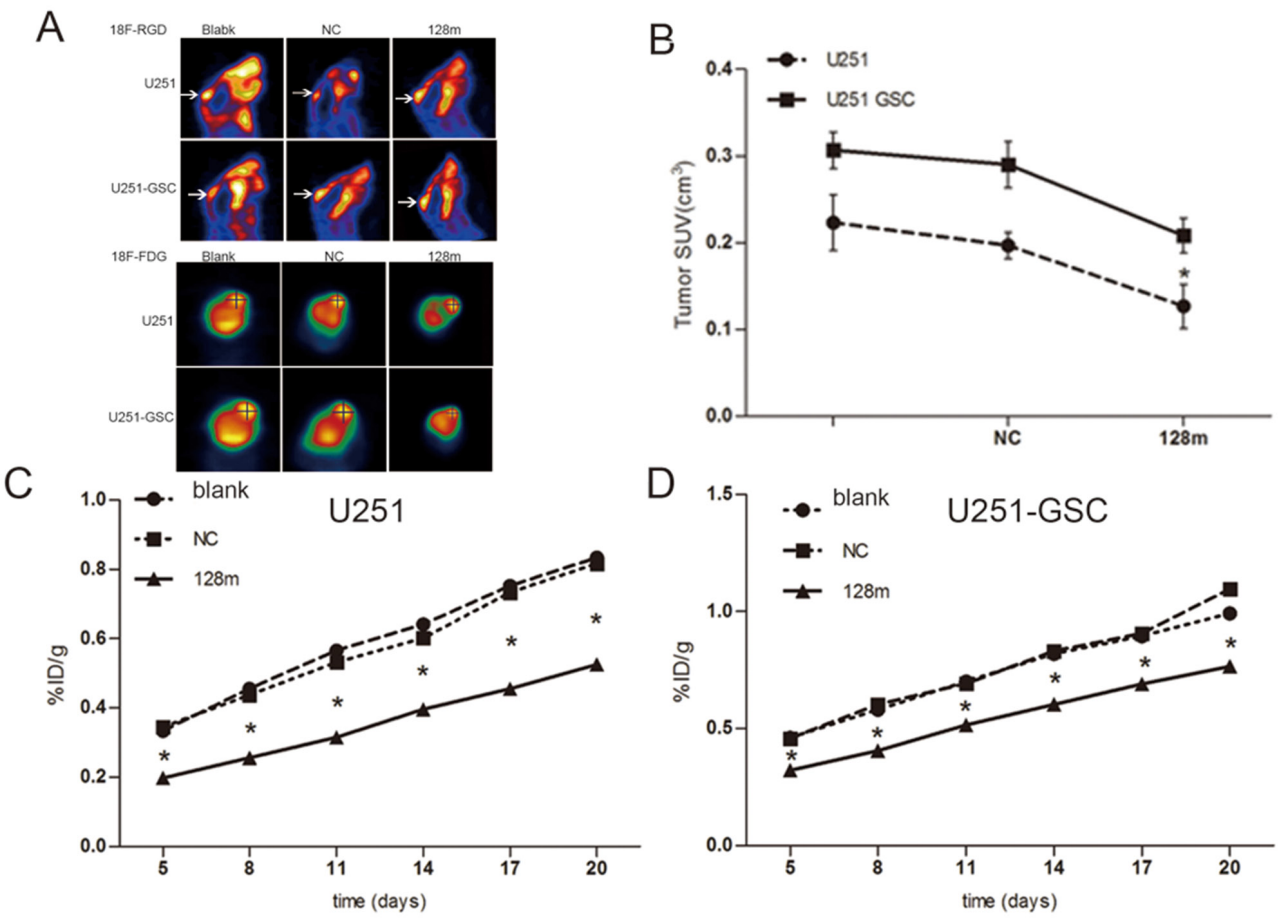
miR128-1 overexpression inhibited the growth of mouse glioma xenografts **A.** After inoculation with U251 cells, transplanted tumor in the mice grew and increased radioactive tracer uptake 18F-RGD (A upper panel) and 18F-FDG (A lower panel) as monitored by MicroPET scanning. **B.** The tumor volume of U251-GSCs group was significantly larger than that of U251 group. The tumor volume of miR128-1 mimic group was smaller than that of NC group. SUV, the mean value of ^18^F-FGD intake. U251 groups: One-way analysis of variance, p<0.01; Bonferroni's Multiple Comparison Test: blank vs NC: t=1.298, p>0.05. *blank vs 128m: t=3.407, p<0.05. * NC vs 128m: t=4.704, p<0.05. U251-GSCs group: One-way analysis of variance, p<0.01; Bonferroni's Multiple Comparison Test, blank vs NC: t=0.9005, p>0.05. blank vs 128m: t=4.412, *p<0.01. NC vs 128m: t=5.313, *p<0.01 *p < 0.05. **C.** Tumor growth curves of U251 groups blank, transfected mock miRNA and miR128-1 as measured by the (%ID/g) value of ^18^F-RGD intake in the intracerebral transplantation tumor. One-way analysis of variance, p<0.0001. Bonferroni's Multiple Comparison Test, blank vs NC: t=0.4317-2.149, p>0.05. blank vs 128m: t=18.7-32.98, *p<0.01. NC vs 128m: t=8.15-31.86, *p<0.01. **D.** Tumor growth curves of U251-GSCs groups with blank, transfected mock miRNA and miR128-1 as measured by the (%ID/g) value of ^18^F-RGD intake in the intracerebral transplantation tumor. One-way analysis of variance, *p<0.0001. Bonferroni's Multiple Comparison Test, blank vs NC: t=0.2465-2.893, p>0.05. blank vs 128m: t=9.379-152.6, *p<0.01. NC vs 128m: t=11.27-152.60, *p<0.01.blank: normal control; NC: transfection with mock miRNA; 128m: miR128-1 mimic. *p < 0.05 (One-way analysis of variance and Bonferroni's Multiple Comparison Test student's t test).

**Figure 7:**
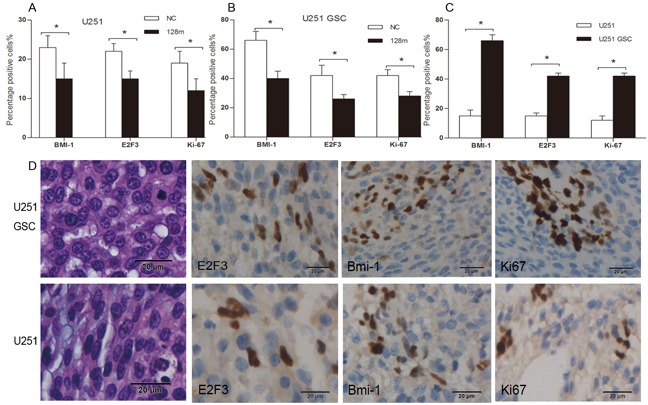
Overexpression of miR128-1 inhibited the growth of nude mice xenografts and decreased the expression of BMI1, E2F3 and Ki67 **A.** The percentage of positive staining cells with the expression of BMI1, E2F3 and Ki67 in U251 xenografts with miR128-1 or miR-NC transfection. The p values are as follows: p = 0.032 (BMI1), 0.036 (E2F3) and 0.041 (Ki67) compared with miR-NC. **B.** The percentage of positive staining cells with the expression of BMI1, E2F3 and Ki67 in U251-GSCs xenografts with miR128-1 or miR-NC transfection. The p values are as follows: p = 0.004 (BMI1), 0.022 (E2F3) and 0.008 (Ki67) compared with miR-NC. **C.** The comparison of the percentage of positive staining cells with the expression of BMI1, E2F3 and Ki67 between U251 and U251-GSCs xenografts. The p values are as follows: p = 0.001 (BMI1), <0.001 (E2F3) and <0.001 (Ki67) U251-GSCs compared with U251. **D.** The expression of BMI1 in cell nuclei and cytoplasm and the expression of E2F3 and Ki67 in tumor cell nuclei of U251 and U251-GSCs xenografts. magnification ×400. *p < 0.05.

miR128-1 overexpression was reported to inhibit the proliferation of glioma cells *in vitro* [19]. To assess whether miR128-1 overexpression reduces glioma growth *in vivo*, we transplanted miR128-1 mimic or miR-NC transfected U251 cells and U251-GSCs into the brains of nude mice to establish mouse glioma xenografts. Weight loss was observed one week after transplantation. The radioactivity data from injected ^18^F-FDG and ^18^F-RGD was collected every three days. Live glioma xenografts growth in the brain was monitored using MicroPET after days 5 (Figure 6A). The tumor volume of U251-GSCs group was significantly larger than U251 group (p<0.01) and tumor volume of miR128-1 mimic group was smaller than that of NC group (p<0.01) (Figure 6B). Interestingly, when compared to U251 group, faster tumor growth and more active ^18^F-FDG uptake were observed in U251-GSCs group. ^18^F-RDG radioactivity was inhibited by miR128-1 overexpression in both U251 cells and U251-GSCs, indication of suppression of tumor growth by miR128-1. Similarly, analysis by ^18^F-RGD showed that miR128-1 overexpression significantly impeded the tumor growth of both U251 and U251-GSCs tumor xenografts (Figure 6C, 6D). Histologically, when compared with the U251 group, large hyperchromatic nuclei with atypical mitosis were more common in miR128-1 transfected U251-GSCs tumors. Moreover, immunohistochemistry showed that the expression of BMI1, E2F3 and Ki-67 was significantly decreased by miR128-1 in comparison to control miRNA in both U251 group (Figure 7A) and U251-GSCs group (Figure 7B). In addition, BMI1, E2F3 and Ki-67 expression was significantly higher in the U251-GSCs group when compared with the U251 group (Figure 7C, and 7D). Our data indicated that miR128-1 expression inhibited the growth of U251 and U251-GSCs mouse tumor xenografts, and that U251-GSCs exhibited enhanced cell proliferation *in vivo*.

## DISCUSSION

In this study, we demonstrated miR128-1 was downregulated in glioma cells and their GSCs when compared with normal brain tissues; however the miR128-1 level in GSCs was restively higher than the corresponding parental glioma cells. Furthermore, we showed that miR128-1 targeted BMI1 and E2F3, and miR128-1 overexpression down-regulated BMI1 and E2F3 in glioblastoma cells both *in vitro* and in mouse tumor xenografts. Most importantly, we discovered that there was decreased DNA methylation in the miR128-1 gene in GSCs in comparison to the corresponding parental glioma cells and treatment of both GBM cells and GSCs with the DNA methylation inhibitors Aza and PBA resulted in miR128-1 up-regulation. Finally, we showed that miR128-1 overexpression impeded the growth of glioblastoma mouse tumor xenografts. Our results demonstrated that aberrant miR128-1 methylation is associated with miR128-1 downregulation in glioma especially in GSCs, suggesting miR128-1 and demethylating agents are promising for glioma treatment.

As a “brain-specific” miRNA, miR128-1 has a tissue-specific expression pattern, and is expressed mainly in neurons rather than in astrocytes [10]. Additionally, miR128-1 is present in terminally differentiated mature neurons, but absent in neural stem cells [20]. miR128-1 is encoded by two distinct intronic genes, miR128-1 and miR128-2, which are embedded in the introns of the R3HDM1 (R3H domain containing 1) and RCS (cyclic AMP-regulated phosphoprotein) genes that are located on human chromosomes 2q21.3 and 3p22.3, respectively [21, 22]. Although most intronic miRNAs depend on host gene expression for transcription and are processed from the same primary transcript, some mammalian intronic miRNAs might be transcribed from their own promoters. In the case of miR128-1, three SNPs are located in the genomic region corresponding to hsa-miR128-1, and the international HapMap project has observed strong geographical genetic variation among different populations in this gene [23]. In miR128-2, the Pol III promoter is found in the 5′-flanking region, it will be interesting to investigate whether the expression of miR128-2 depends on its host gene ARPP-21 [24, 25]. Aberrant miR128-1 expression has been observed in many malignancies. Although miR128-1 downregulation has been reported in GBM and neuroblastoma, miR128-1 upregulation has also been reported in acute myeloid leukemia and letrozole-resistant breast cancer cell lines [11]. These findings indicate that miR128-1 can function as either an oncogenic or a tumor-suppressive miRNA, depending on the specific tumor type. In glioma tissues, miR128-1 expression was found to be downregulated when compared with normal human brain tissues [26, 27]; however, the mechanism of miR128-1 deregulation in glioma tissues remains to be determined. In the present study, we provided direct evidence that epigenetic methylation of miR128-1 is one of the mechanisms underlying miR128-1 downregulation in glioma.

The heterogeneous nature of glioma cells is believed to contribute to their chemotherapy resistance and patient relapse after therapy [28]. Although the hierarchical structure of gliomas and the models of heterogeneity are controversial, the presence and contribution of the tumor-initiating GSCs to heterogeneity has been well established [29, 30]. Interestingly, we found that ectopic miR128-1 expression lead to higher overall miR128-1 expression in GSCs when compared to glioma cell lines, suggesting an unknown mechanism promoting miR128-1 expression or stabilizing miR128-1 in GSCs. To test this hypothesis, we treated glioma cells and their GSCs with Aza and PBA, a potent DNA methylation inhibitor and a histone deacetylase inhibitor, respectively. After Aza and PBA treatment, miR128-1 upregulation was observed in both glioma cells and their GSCs. Similar to the miR128-1 mimic transfection, inhibition of DNA methylation induced higher miR128-1 expression in GSCs. It is believed that Aza and PBA may reduce DNA methylation levels and then open chromatin structures, thereby inducing the re-expression of epigenetically silenced genes [31, 32]. Indeed, inhibition of DNA methylation by Aza and PBA resulted in elevated expression of miR128-1 in both glioma cells and GSCs. Furthermore, we identified three DNA methylation sites in miR128-1 by performing BSP sequencing. One of three CpG islands in the miR128-1 gene was methylated in U251-GSCs while all three were methylated in U251 cells. These data indicate that DNA methylation downregulates miR128-1 expression in glioma cells and decreased DNA methylation contributes to the relatively increased expression of miR128-1 in GSCs compared with the parental glioma cells.

Several studies have explored miR128-1 target genes that may potentially play a role in the regulation of cell differentiation and self-renewal [33]. Of the stem cell-related genes, BMI1 is one of the most important miR128-1 targets. BMI1 is a component of the polycomb repressor complex (PRC), and suppresses the expression of key target genes through chromatin modification. BMI1 also plays a role in stem cell renewal and serves as a neural stem cell and glioma maintenance factor [17, 18, 34]. Consistent with the observations in prostate cancer [16], our study found that miR128-1 negatively regulated BMI1 expression in glioma cells through its predicted miR128-1 binding site. Additionally, we found that E2F3, a transcription factor that regulates cell cycle progression, was also a direct target of miR128-1. Accordingly, miR128-1 overexpression resulted in reduced expression of both BMI1 and E2F3 in glioma cells and GSCs. These results indicate that one of the mechanisms by which miR128-1 regulates cell differentiation and self-renewal is via the targeting of BMI1 and E2F3.

In addition, our results demonstrated that miR128-1 inhibited the growth of glioma. miR128-1 overexpression inhibited the sphere-forming activities, proliferation and migration of cultured glioma cells. In the *in vivo* study, we employed miroPET scanning to monitor tumor growth in mice without sacrificing mice. ^18^F-RGD and ^18^F-FDG are radioactive tracers that are a tumor metabolism markers and can be used to observe live tumor growth *in vivo*. Interestingly, MicroPET scans of glioma xenograft-bearing mice showed lower activity for both tracers in the brains of miR128-1 overexpressing U251 or U251-GSCs cells, indicating the tumor growth was inhibited in mice transplanted with miR128-1 overexpressed cells. It has been well accepted that ultimate cure of cancer depends on the elimination of cancer stem cells.

In conclusion, we found that miR128-1 level was relatively higher in GSCs than glioma cells, DNA methylation negatively regulated miR128-1 expression in glioma cells and overexpression of miR128-1 suppressed the growth of glioma cells both *in vitro* and *in vivo*. Our results confirmed the published data that miR-128 inhibits glioma proliferation and self-renewal via targeting Bmi-1 [8] and revealed a new mechanism by which miR128-1 is deregulated in glioma. Our findings suggest that miR128-1 and DNA demethylating agents are promising for anti-glioma therapy at least in part by eliminating GSCs.

## MATERIALS AND METHODS

### Cell culture and scanning electron microscopy (SEM) analysis

Human glioma U87 and U251 cells were obtained from Shanghai Life Sciences Research Institute Cell Resources Center and maintained in high glucose DMEM (Invitrogen, Grand Island, NY, USA) containing 10% fetal bovine serum (FBS; Sigma Aldrich, St. Louis, MO, USA). All cells were maintained in 5% CO_2_ at 37°C. The cells were grown and differentiated in serum- free DMEM/F12 medium with 2% B27 (Invitrogen), 10 ng/mL human recombinant leukemia inhibitory factor (PeproTech, Hamburg, Germany), 20 ng/mL basic fibroblast growth factor (PeproTech), 20 ng/mL epidermal growth factor (PeproTech), 100 units/mL penicillin (NCPC, Shijiazhuang, China) and 100 μg/mL streptomycin (NCPC). Gliospheres were counted under a light microscope after culturing for 7 - 10 days. The characteristics expression of CD133 and nestin but negative expression of GFAP of tumor cells of “stem-like” cell subpopulation was assessed by immunofluorescence techniques. For SEM analysis, cells were cultured on the slide chamber before fixing with 2.5% glutaraldehyde and 1% osmic acid. After dehydration with gradient acetone, the slides were incubated with isoamyl acetate and the spray-dried samples were examined under a scanning electron microscope (FEI, Hillsboro, OR, USA).

### GSCs Matrigel colony forming assay and differentiation assay

GSCs-enriched cells were harvested, suspended in serum- free DMEM/F12 medium with 0.2% Matrigel (BD Biosciences) and overlaid onto 0.5 mm thick bottom Matrigel in a 6-well plate at 30 cells/well. The GSCs colonies (> 10 cells) were counted under a light microscope after culturing for 10 days. For differentiation assay, GSCs-enriched cells were harvested, suspended in DMEM medium and cultured till all become adherent growth of tumor cells. The differentiated cells were confirmed by the immune phenotypic expression of GFAP but not CD133 by flow cytometry (BD Biosciences).

### Vector construction

A 377-bp fragment of miR128-1 gene was amplified by PCR from genomic DNA isolated from human brain tissue and cloned into vector pcDNA3.1 (Promega, Madison, WI, USA). A 3′-untranslated region (UTR) luciferase reporter vector was constructed by ligating a fragment of the BMI1 and E2F3a 3′-UTR encompassing the miR128-1 binding sequence into the pcDNA3.1-luc vector (Promega). The predicted miR128-1 target site CACTGTG was converted to ACGACAC by site-directed mutagenesis. Primer sets (5′ - 3′) were as follows: miR128-1 cloning, GATTTTAGGTTTACAAAGCCCTAGCTGT and CTAATCCCTATTTCTGAGTATGATGCATGA; Mutagenic primers for BMI1 were TAATGCATTCTA TGTAGCCATGTTGTTGT and GAATAACGATTTC TTGCATATTTAG; Mutagenic primers for E2F3 were TAAATATGCAAGAAATCGTTATTCACAAC and AACAT GGCTACATAGAATGCATTA; BMI1 reporter construct, TATATCTAGATTCTTGTTATTACGCTGTTTTG and AGATTCTAGAATGTCATATACCAATATGGC; E2F3 reporter construct, AAACAATGCCAGGGTGTCTC and TAGCCATTTCGTGTGTGAGC.

#### Western blot analysis

Total protein was separated on a precast 4% to 15% sodium dodecyl sulfate polyacrylamide gel (Invitrogen) and transferred onto polyvinylidene fluoride membrane, followed by probing with antibodies for BMI1, E2F3, and GAPDH (loading control). Detection of HRP-conjugated antibodies was performed. Protein bands were visualized by an ECL plus chemiluminescence (Beyotime, Haimen, China). Densitometric analysis of protein bands was performed via using Image J software.

### Real-time PCR

Total RNA was extracted using mirVana miRNA Isolation Kit (Ambion, Life Technologies) and treated with RNase-free DNase (Qiagen). Mature miR128-1 expression analysis was carried out using miRNA TaqMan quantitative reverse-transcription PCR assay (Applied Biosystems, Life Technologies) normalized to U6 snRNA (Applied Biosystems). PCR reactions were performed and analyzed using the ABI 7300 system. BMI1 and E2F3 mRNA expression was measured using the SYBR Green PCR System (Applied Biosystems) with human BMI1, E2F3 and Actin primers. Primer sets (5′ - 3′) were as follows: BMI1: CACCAGAGAGATGGACTGACAA and AGGAAACTGTGGATGAGGAGAC; E2F3: ACAAACAACCAAGACCACAATG and GGGAGGC AGTAAGTTCACAAAC; Actin: AGTGTGACGTGG ACATCCGCAAAG and ATCCACATCTGCTGGAAGG TGGAC. The relative expression was calculated using the 2^−Δ ΔCT^ method.

### Cell transfection and drug treatment

Transfection of miR128-1 mimic oligonucleotide (200nM) and negative control (NC) (GenePharma, Shanghai, China) was performed using Lipofectamine 2000 (Invitrogen) according to the manufacturer's protocol. Twenty-four hrs after transfection, cells were harvested and RNA was extracted for real-time PCR (RT-PCR) analysis. For the methylation inhibition experiments, glioma cells were seeded at 5 × 10^5^ cells per 10 cm dish for 24 hrs before treatment with Aza (1 μM or 3 μM, Sigma-Aldrich) or PBA (1 mM or 3 mM, Sigma-Aldrich). For the combination treatment, cells were treated with Aza for 24 hrs, followed by PBA treatment for an additional 5 days.

### Luciferase assay

U251 and U87 cells were plated in 24-well plates for 24 hrs, and then co-transfected with miR128-1 or pcDNA3.1-luc vector containing wild-type or mutant 3′UTR using lipofectamine 2000. Luciferase assays were performed 48 hrs after transfection using the Dual Luciferase Reporter Assay System (Promega) according to the manufacturer's instructions.

### Flow cytometry analysis

Cells were trypsinized and Accutase (Millipore) was used to dissociate GSCs. After washing with PBS, cells were centrifuged and stained with primary antibody, followed by incubation with FITC or PE conjugated secondary antibodies. The cells were then subjected to flow cytometry analysis on BDAria FACS machine (BD Biosciences, San Jose, CA, USA) and the data was analyzed using CellQuest software (BD Biosciences). Flow cytometry antibodies included: anti-human Fc-receptor (Catalog #130-095-979, Miltenyi Biotec, Bergisch Gladbach, Germany), anti-human CD133/2-PE (Catalog #130-080-901, Miltenyi Biotec), GFAP (Catalog #60048; STEMCELL Technologies, Vancouver, Canada), Nestin Antibody (S1409, Abgent, San Diego, USA), BMI1 (ab14389; Abcam, Cambridge, UK), E2F3 (LS-C87464; Lifespan-Bioscience Inc., Seattle, WA, USA), FITC or PE-conjugated goat anti-rabbit (ab6717; Abcam) and FITC or PE-conjugated goat anti-mouse mouse (ab6785; Abcam) secondary antibodies.

### Cell proliferation assay

2 × 10^3^ cells were seeded in 96-well plates. Cell Counting Kit-8 solution (10 μL per well; Dojindo Laboratories, Kumamoto, Japan) was added and incubated for additional 4 hrs. Optical density was determined with a spectrophotometer by measuring the absorption of the excitation wave at 450 nm and the emission wave at 600 nm (Spectramax 190; Molecular Devices, Sunnyvale, CA, USA).

### Wound healing assay

Cells were seeded in 6-well plates and incubated to 80% confluence. The cell monolayer was gently scraped with a 10-μL pipette tip and washed three times with PBS solution and incubated at 37°C. The scraped cell monolayer was photographed at various time intervals. Images were acquired using computer-assisted microscopy and the wound width was measured at various time points.

### Cell invasion and migration assays

2.5 × 10^5^ cells suspended in 250 μL serum-free DMEM were seeded in the top chambers of 24-well transwell plates (Corning Inc., Corning, NY, USA) coated with 30 μL Matrigel (BD Biosciences). The bottom chambers of the transwell plates were filled with 600 μL DMEM containing 10% FBS. Cells were allowed to migrate for 24 - 48 hrs at 37°C. After migration, cells in the top chambers were removed using a cotton swab and the cells that migrated to the bottom chambers were fixed in 4% paraformaldehyde (PFA; Sigma-Aldrich) and stained with Crystal Violet. The fixed and stained cells were counted in five independent fields under a light microscope. At least three chambers were counted for each experiment.

### Bisulfite sequencing PCR

miR128-1 DNA methylation was evaluated using Bisulfite sequencing PCR (BSP). CpG islands databases (www.ebi.ac.uk/emboss/cpgplot) were used to identify the CpG islands (CGIs) spanning the miR128-1 gene. Primers targeting all three CGIs (sequence number 26, 32 and 72) were designed using Methprimer software. Genomic DNA isolation and bisulfite conversion were performed as described previously [35]. Bisulfite-converted genomic DNA, which converts only unmethylated cytosines to uracils, was amplified using strand-specific primers followed by digestion with methylation sensitive enzymes. The primers used for miR128-1 CGI amplification were (5′ - 3′): GGTTTTGTTTTTGAGTTGTTGG and AACAAATATTAACACCTTCATACAACA. DNA methylation levels were determined by bisulfite genomic sequencing using an ABI Prism 377 (Applied Biosystems).

### Animal experiments

All animal experiments were approved by Institutional Animal Care and Use Committee of Anhui Provincial Hospital affiliated to Anhui Medical University (No. LLSC2013014). Athymic/nude immunocompromised mice were purchased from Nanjing University (Nanjing, China) and breeding colonies were maintained in our animal facility under standard conditions. Intracranial transplantation of glioma cells or GSCs into athymic/nude immunocompromised mice was performed as previously described [36]. Briefly, six to eight week old male nude mice were divided into six groups (five mice per group). These groups included the control group, miR-NC group and miR128-1 group for both the U251 and the U251-GSCs cell lines. After pre-transplant preparation of the recipient mice and anesthesia with 10% chloral hydrate, isolated U251-GSCs or U251 cells (10^6^) were transplanted into the right frontal lobes of the recipient mice to establish the xenograft model. The weight change of each animal was measured daily. A miroPET scan was performed on the tenth day after tumor cell transplantation via tail vein administration of ^18^F-FDG or ^18^F -RGD. The mice were monitored every day. Cancer cachexia symptoms, such as weight loss > 20%, limbs paralysis or movement disorder, lethargy, a hunched posture and growing hair, were set as the experimental endpoint. When they displayed obvious cancer cachexia symptoms, the mice were sacrificed, and the brains were harvested for histological analysis. On day 20, all alive U251 and U251-GSCs recipient mouse brains were harvested for histological analysis. Prior to harvesting, cardiac perfusion with PBS followed by 4% PFA perfusion was performed. Brain tumor xenografts were fixed using 4% PFA overnight, post-fixed in 70% ethanol, embedded with paraffin and sectioned to 4 μM for subsequent histological and immunohistochemistry analysis.

### Immunohistochemistry staining

Immunohistochemistry (IHC) was performed using the formalin-fixed and paraffin-embedded brain tissues. The primary antibodies used were E2F3 (1:100 dilution; Abcam), BMI1 (1:100 dilution; Abcam) and Ki67 (1:100; Roche, Basel, Switzerland). A universal DAB detection kit (Roche) was used to stain antibody bound tissues. Hematoxylin/eosin was used to counterstain all slides. Stained sections were examined under a light microscope and the positive cells in five high power fields (10×40) were counted and averaged to attain each sample's final score.

### Statistical analysis

Statistical analyses were performed with SPSS for windows (SPSS Inc., Chicago, IL, USA). Independent samples were compared using two-tailed unpaired t test. One-way analysis of variance was performed for the comparison among multiple groups while Bonferroni's Multiple Comparison Test was performed to compare the difference between two groups. All statistical results from the quantitative analysis of the *in vitro* experiments are presented as means ±SEM or ±SD, as specified in the figure legends. p values < 0.05 were considered statistically significant.
